# Genetic alteration patterns and clinical outcomes of elderly and secondary acute myeloid leukemia

**DOI:** 10.1002/hon.2656

**Published:** 2019-08-20

**Authors:** Shi‐Yang Wang, Wen‐Yan Cheng, Yuan‐Fei Mao, Yong‐Mei Zhu, Fu‐Jia Liu, Ting‐Ting Ma, Yang Shen

**Affiliations:** ^1^ Shanghai Institute of Hematology, Department of Hematology Ruijin Hospital Affiliated to Shanghai Jiao Tong University School of Medicine Shanghai China

**Keywords:** acute myeloid leukemia, elderly, genetic, prognosis, secondary

## Abstract

To illustrate the clinical and genetic features of elderly and secondary acute myeloid leukemia (AML) patients, we compared 145 elderly AML (e‐AML) and 55 secondary AML (s‐AML) patients with 451 young de novo AML patients. Both e‐AML and s‐AML patients showed lower white blood cell (WBC) and bone marrow (BM) blasts at diagnosis. *NPM1*, *DNMT3A*, and *IDH2* mutations were more common while biallelic *CEBPA* and *IDH1* mutations were less seen in e‐AML patients. s‐AML patients carried a higher frequency of *KMT2A*‐AF9. In treatment response and survival, e/s‐AML conferred a lower complete remission (CR) rate and shorter duration of event‐free survival (EFS) and overall survival (OS) compared with young patients. In multivariate analysis, s‐AML was an independent risk factor for OS but not EFS in the whole cohort. Importantly, intensive therapy tended to improve the survival of e/s‐AML patients without increasing the risk of early death, and hematopoietic stem cell transplantation (HSCT) could rescue the prognosis of s‐AML, which should be recommended for the treatment of fit patients.

## 1 INTRODUCTION

Acute myeloid leukemia (AML) is a group of biological and clinical heterogeneous hematologic malignancies, whose prognosis is strongly associated with underlying genetic alterations and clinical factors, especially the history of antecedent hematological diseases or cytotoxic treatment, which is called secondary acute myeloid leukemia (s ‐ AML). In addition, age is another important clinical feature, which exerts negative effect on the disease. More importantly, AML is increasingly considered as a senile disease, which was reported of a median age of 66 in the United States^1^ and 71 in Sweden.^2^ With the development of high dose chemotherapy, hematopoietic stem cell transplantation (HSCT) and even tailored therapy, the treatment outcome of AML has improved significantly in the last decades; however, the prognosis of elderly AML (e‐AML) and s‐AML remains dismal. Both elderly and secondary AML (e/s‐AML) patients present with increased age, poor performance status, more comorbidities, depleted hematopoietic reserves, and more importantly, the disease‐associated factors, such as unfavorable cytogenetic and molecular abnormalities, leading to insufficient treatment and poor treatment outcome.^3^, ^4^, ^5^ It was reported that e/s‐AML patients harbored less favorable cytogenetics such as *CBF*‐rearrangements but more unfavorable cytogenetics especially abnormalities involving 5 or 7 chromosome at diagnosis.^2^, ^6^ Genetic landscape of AML has been widely studied in the past decades, however, most of previous studies focused on de novo AML especially those patients with young age,^7^, ^8^ while reports regarding genetic alterations and their prognostic significance in e/s‐AML are still rare.

More importantly, the treatment of e/s‐AML remains controversial. Various modalities, such as hypomethylation agents as exemplified as decitabine and azacitidine, and low doses chemotherapy were tried in this group of patients; however, no therapeutic regimen was proved to be significantly superior to traditional chemotherapy. To some extent, the treatment decision was strongly dependent on the fitness of AML patients.

In this study, we examined genetic alterations and post‐treatment minimal residual diseases (MRD) in order to illustrate their distribution and prognostic impact in e/s‐AML and to provide treatment recommendations for those patients.

## 2 METHODS

### 2.1 Patients

From January 2013 to December 2017, a total of 651 adult patients (18 years old or above) with newly diagnosed non‐M3 AML at Shanghai Institute of Hematology (SIH) were executively enrolled in this study, among which, 55 patients were diagnosed as s‐AML (34 patients had an antecedent hematological disease [AHD‐AML] and 21 patients were diagnosed as therapy‐related AML [t‐AML]). Cytogenetic risk stratification was based on 2017 European LeukmiaNet (ELN) recommendations.^9^

This study was approved by the ethic committee of Ruijin Hospital. All patients had given informed consent for both treatment and cryopreservation of bone marrow (BM) and peripheral blood according to the Declaration of Helsinki.

### 2.2 Treatment protocols

For young de novo patients (younger than 60 years old), standard intensive “3 + 7” induction regimens (idarubicin 10‐12 mg/m^2^ or daunorubicin 45‐60 mg/m^2^, D1‐3; cytarabine 100 mg/m^2^ D1‐7) were given as the initial induction therapy. If CR was achieved, four cycles of high‐dose cytarabine (2 g/m^2^) was given as consolidation. For e‐AML (60 years and older) and s‐AML patients, the treatment was mainly decided by the physician in consideration of the fitness of patients and risk of disease. Fit patients received treatment similar to young patients but reduced cycles of consolidation to 2 cycles of high‐dose cytarabine; unfit patients received “3 + 7” regimens with reduced dose, hypomethylation treatment or palliative treatment according to the physician's decision.

### 2.3 Molecular events and MRD

Genetic alterations including *FLT3*‐ITD/TKD, *KMT2A*‐PTD, *NPM1*, *NRAS*, *CKIT*, *CEBPA*, *DNMT3A*, *IDH1*, *IDH2*, *RUNX1‐RUNXT1T1*, *CBFβ‐MYH11*, *KMT2A* rearrangements were detected as previously reported.^10^ Bone marrow aspirate samples were processed according to the standard procedure of our institution as previously reported.^11^ Detection of MRD after induction therapy was based on leukemia‐associated immunophenotype (LAIP) at diagnosis and performed by ten‐color multiparametric flow cytometry. MRD was considered positive when leukemia cells were greater than or equal to 0.01%.

### 2.4 Statistical analyses

Complete remission (CR) was defined by the criteria of the International Working Group.^12^ Early death (ED) was defined as death within 30 days after diagnosis. Overall survival (OS) was measured from the date of disease diagnosis to death from any cause, and patients alive at last follow‐up were censored. Event‐free survival (EFS) was defined as the time from diagnosis to the date of relapse (if achieved CR) or death from any cause, whichever occurred first, with patients still alive censored at the date of last follow‐up. Patients who received HSCT were censored at the time of HSCT to eliminate its impact on EFS and OS. The Kaplan‐Meier method was used to calculate the distribution of OS and EFS. A log‐rank test was performed to compare the difference in survival time. Multivariate analyses were conducted by using binary logistic regression for CR and ED, and Cox proportional hazard model for OS and EFS. All of the above statistical procedures were carried out by using the SPSS Version 24.0 statistical software package.

## 3 RESULTS

### 3.1 Characteristics of patients

The baseline characteristics of patients were shown in Table 1. Patients with s‐AML presented female predominance (*P* = .041), most of whom had a previous history of breast carcinoma (43%). Older age (*P* < .001), lower white blood cell (WBC) count (*P* = .031), hemoglobin (HB, *P* = .004), and BM blasts (*P* < .001) were observed in s‐AML as compared with young patients. Similarly, elderly patients showed lower WBC (*P* = .036) and BM blasts (*P* = .009) at diagnosis. In WHO subtype distribution, higher frequency of pure erythroid leukemia was seen in s‐AML (*P* = .013). Both e‐AML and s‐AML patients received less intensive induction, but more hypomethylation treatment and palliative treatment than younger patients (all *P* < .001).

**Table 1:** Clinical characteristics of AML patients

			de novo AML		
Factor	s‐AML, N = 55	*P* a	Young, N = 451	Elderly, N = 145	*P* a
Age, y		<.001			<.001
Median	57		43	65	
Range	21‐77		18‐59	60‐81	
Male gender, n (%)	22 (40.0)	.041	246 (54.5)	76 (52.4)	.654
WBC count, ×10^9^/L		.031			.036
Median	6.8		16.83	10.56	
Range	0.8‐144.1		0.77‐419.9	0.5‐241.94	
HB, g/L		.004			.338
Median	69		85	82	
Range	34‐143		30‐171	15‐142	
PLT count, ×10^9^/L		.074			.099
Median	60		41	44	
Range	3‐752		2‐1726	3‐512	
BM blasts, %		<.001			.009
Median	39.5		69	60.5	
Range	16.5‐95		7‐98.5	17.5‐96.5	
WHO category, n (%)					
AML with recurrent genetic abnormalities					
AML with t(8;21)(q22;q22.1); *RUNX1‐RUNX1T1*	2(3.6)	.042	59(13.1)	10(6.9)	.043
AML with inv(16)(p13.1q22) or t(16;16)(p13.1;q22); *CBFB‐MYH11*	0(0)	.112	28(6.2)	4(2.8)	.109
AML with t(9;11)(p21.3;q23.3); *MLLT3‐KMT2A*	4(7.3)	.013	6(1.3)	1(0.7)	.857
AML with inv(3)(q21.3q26.2) or t(3;3)(q21.3;q26.2); *GATA2, MECOM*	0(0)	1	1(0.2)	1(0.7)	.428
Provisional entity: AML with *BCR‐ABL1*	0(0)	1	1(0.2)	0(0)	1
AML with mutated *NPM1*	7(12.7)	.436	76(16.9)	39(26.9)	.008
AML with biallelic mutations of *CEBPA*	3(5.5)	.048	69(15.3)	11(7.6)	.018
AML, NOS					
AML without maturation	0(0)	1	1(0.2)	2(1.4)	.148
AML with maturation	0(0)	.45	12(2.7)	6(4.1)	.532
Acute myelomonocytic leukemia	9(16.4)	.837	69(15.3)	18(12.4)	.392
Acute monoblastic/monocytic leukemia	6(10.9)	.387	69(15.3)	25(17.2)	.577
Pure erythroid leukemia	4(7.3)	.013	6(1.3)	2(1.4)	1
Not classified	20(36.4)	<.001	54(12.0)	26(17.9)	.067
Therapy					
Intensive induction	23 (41.8)	<.001	422 (93.6)	79 (54.5)	<.001
Hypomethylation	12 (21.8)	<.001	5 (1.1)	15 (10.3)	<.001
Palliative treatment	20 (36.4)	<.001	24 (5.3)	51 (35.2)	<.001

Abbreviation: AML, acute myeloid leukemia; BM, bone marrow; HB, hemoglobin; NOS, not otherwise specified; PLT, platelet; WBC, white blood cell; WHO, The World Health Organization.

a All compared with young patients.

### 3.2 Cytogenetic and genetic alterations

In cytogenetic classification, elderly patients had a significantly higher proportion of intermediate risk cytogenetics (*P* = .011). Favorable cytogenetic alterations were less frequent in both elderly and secondary patients (*P* = .008 and.014, respectively) as compared with young patients.

With regard to genetic abnormalities, the incidence of CBF leukemia was significantly lower in e/s‐AML patients as compared with young patients (7.1% vs 14.7%, *P* = .013 for *RUNX1‐RUNX1TI* and 2.6% vs 7.4%, *P* = .038 for *CBFβ‐MYH11*). A higher frequency of *NPM1* (*P* = .003), *DNMT3A* (*P* = .015), and *IDH2* (*P* = .004) mutations, but lower frequency of biallelic *CEBPA* (Bi*CEBPA*, *P* = .029) and *IDH1* (*P* = .038) mutations were observed in elderly patients. In addition, s‐AML patients carried *KMT2A*‐AF9 (*P* = .007) more frequently when compared with young de novo patients (Table 2).

**Table 2:** Cytogenetic and genetic alteration patterns of acute myeloid leukemia (AML) patients

			de novo AML		
Variable Number/Total (%)	s‐AML, N = 55	*P* a	Young, N = 451	Elderly, N = 145	*P* a
Cytogenetics					
Favorable	3/49 (6.1)	.014	84/404 (20.8)	13/126 (10.3)	.008
Intermediate	38/49 (77.6)	.076	262/404 (64.9)	97/126 (77.0)	.011
Unfavorable	8/49 (16.3)	.712	58/404 (14.4)	16/126 (12.7)	.639
Genetic Alterations					
*RUNX1‐RUNX1T1*	2/46 (4.3)	.052	59/401 (14.7)	10/122 (8.2)	.063
*CBFβ‐MYH11*	0/37 (0)	.170	28/378 (7.4)	4/114 (3.5)	.139
*FLT3*‐ITD	4/44 (9.1)	.416	54/402 (13.4)	18/124 (14.5)	.759
*FLT3*‐TKD	3/44 (6.8)	.932	21/400 (5.2)	6/124 (4.8)	.856
*KMT2A*‐fusion	5/44 (11.4)	.193	21/400 (5.2)	6/123 (4.9)	.870
*KMT2A*‐AF9	4/44 (9.1)	.007	6/400 (1.5)	1/123 (0.8)	.896
*KMT2A*‐PTD	2/44 (4.5)	.956	24/399 (6.0)	9/123 (7.3)	.604
*NPM1*	7/44 (15.9)	.618	76/400 (19.0)	39/123 (31.7)	.003
*CKIT*	2/41 (4.9)	.354	42/387 (10.9)	10/118 (8.5)	.457
*NRAS*	7/44 (15.9)	.669	54/398 (13.6)	18/124 (14.5)	.789
*BiCEBPA*	3/45 (6.7)	.070	69/403 (17.1)	11/122 (9.0)	.029
*DNMT3A*	7/46 (15.2)	.373	43/397 (10.8)	24/125 (19.2)	.015
*IDH1*	2/23 (8.7)	.971	28/314 (8.9)	2/87 (2.3)	.038
*IDH2*	1/22 (4.5)	.996	22/314 (7.0)	15/87 (17.2)	.004

a All compared with young patients.

As for the association between genetic abnormalities and clinical features, *NPM1* mutations were associated with higher WBC in elderly patients (*P* = .037). Moreover, s‐AML patients with *KMT2A*‐AF9 were prone to having higher BM blasts (*P* = .068) (Table S1).

#### 3.2.1 Treatment responses

In total cohort, CR rate and ED rate were 76.1% and 10.5%, respectively. Both s‐AML and e‐AML patients conferred reduced CR rate as compared with young patients (s‐AML vs young: 58% vs 83%, *P* < .001; e‐AML vs young, 60.7% vs 83%, *P* < .001). Additionally, a higher frequency of ED (e‐AML vs young: 16.6% vs 8%, *P* = .003) was observed in e‐AML (Table 3). In order to find significant factors that can independently predict ED and CR, we conducted univariate and multivariate analyses (Tables S1 and 4).

**Table 3:** Treatment responses

			de novo AML		
Factor	s‐AML, N = 55	*P* a	Young, N = 451	Elderly, N = 145	*P* a
CR status		<.001			<.001
CR, % (n)	58 (29)		83 (356)	60.7 (82)	
Missing/unknown	5		22	10	
Early death		.172			.003
Yes, % (n)	14.5 (8)		8 (36)	16.6 (24)	
Missing/unknown	0		2	0	
MRD		.039			.819
<0.01%, % (n)	17.4 (4)		39.1 (101)	37.5 (21)	
Missing/unknown	32		193	89	

Abbreviation: AML, acute myeloid leukemia; CR, complete remission; MRD, minimal residual disease.

a All compared with young patients.

Among patients achieving CR, 258 young, 56 elderly, and 23 secondary AML patients had a definite LAIP feature before treatment, and the MRD of whom could be monitored. The frequency of positive MRD was higher in s‐AML than in young patients (*P* = .039, Table 3). When e‐AML and s‐AML patients were put together, those who were treated with intensive therapy had a higher CR rate (74.2% vs 44.3%, *P* < .001) and tended to have a lower incidence of positive MRD (60.8% vs 82.1%, *P* = .051) than those treated with other therapy categories. In addition, e/s‐AML patients receiving intensive therapy tended to have a lower ED rate than those who were treated with palliative treatment (12.7% vs 22.5%, *P* = .090).

### 3.3 Impact of prognostic factors on survival

**Figure 1:**
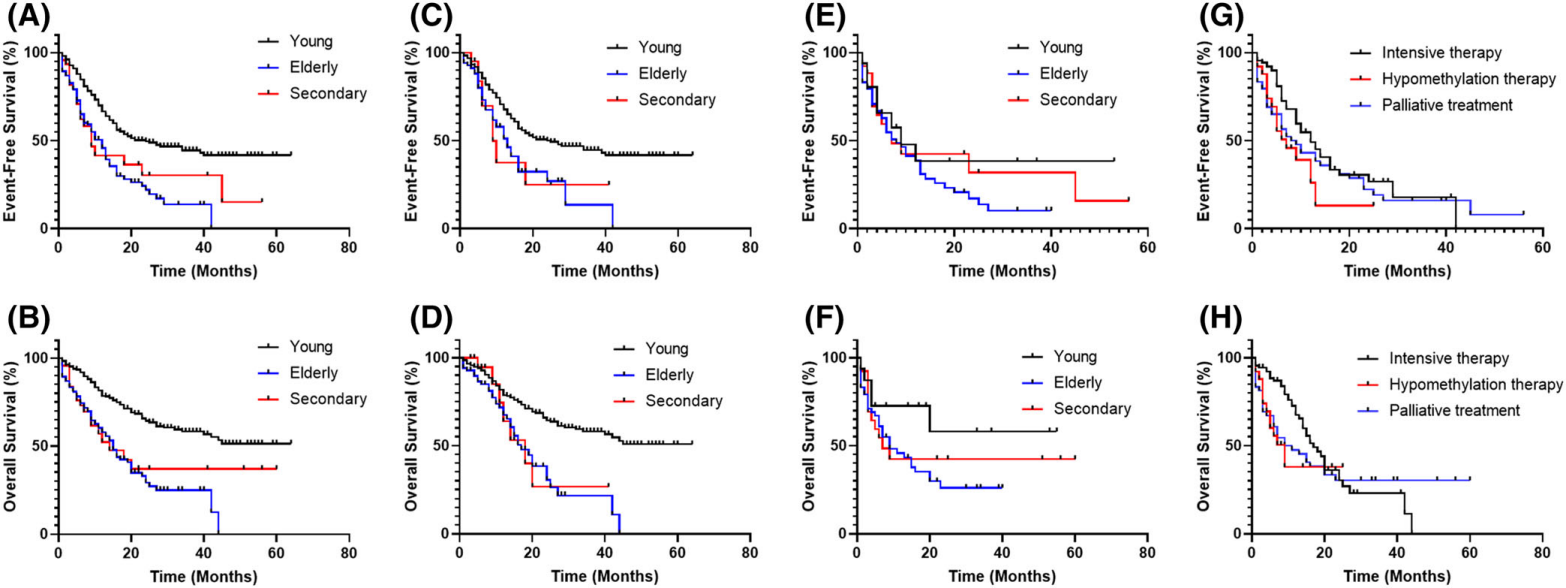
Kaplan‐Meier curves for probability of event‐free survival and overall survival. A,B, Event‐free survival and overall survival for all young, elderly, and secondary acute myeloid leukemia (AML) patients. C,D, Event‐free survival and overall survival for young elderly and secondary AML patients treated with intensive therapy. E,F, Event‐free survival and overall survival for young, elderly, and secondary AML patients treated with less intensive therapy. G,H Event‐free survival and overall survival for patients received intensive therapy, hypomethylation therapy and palliative treatment in elderly and secondary AML group

The median follow‐up in all patients was 27 months (range, 0‐66 months). Generally, e/s‐AML patients had inferior EFS and OS compared with young patients (elderly vs young: 9 vs 18 months for EFS, *P* < .001, and 12 vs 44 months for OS, *P* < .001; s‐AML vs young: 7 vs 18 months for EFS, *P* < .001, and 11 vs 44 months for OS, *P* < .001, respectively) (Figure 1A,B). In the stratification of patients who received intensive therapy, e/s‐AML patients also conferred shorter EFS and OS than young patients (elderly vs young: 12 vs 20 months for EFS, *P* < .001, and 15 vs 44 months for OS, *P* < .001; s‐AML vs young: 9 vs 20 months for EFS, *P* = .022, and 14 vs 44 months for OS, *P* = .026, respectively) (Figure 1C,D). However, there was no difference in EFS and OS between young and e/s‐AML patients who received less intensive therapy (Figure 1E,F). In elderly patients, the median EFS and OS were significantly longer in patients who received intensive therapy, as compared with other treatment modalities (12 vs 6 months for EFS, *P* = .025, and 15 vs 6 months for OS, *P* = .04, respectively). A similar tendency was observed in s‐AML (9 vs 5 months for EFS, *P* = .35 and 14 vs 5 months for OS, *P* = .149). Combining e‐AML and s‐AML patients together, patients receiving intensive therapy were prone to having a longer EFS and OS than those treated with decitabine‐based hypomethylation therapy (10 vs 6 months for EFS, *P* = .093, and 15 vs 7 months for OS, *P* = .067, respectively) and palliative treatment (10 vs 6 months for EFS, *P* = .048, and 15 vs 6 months for OS, *P* = .057, respectively) (Figure 1G,H). Univariate analysis for EFS and OS was shown in Table S3. In order to explore the prognostic significance of increased age and s‐AML after accounting for other recognized prognostic factors, we conducted multivariate analysis (Table 4). In whole cohort, s‐AML relative to de novo AML was an independent risk factor for OS (*P* = .009), while it was not associated with EFS. Notably, the independent prognostic impact of s‐AML on OS was lost when HSCT was not regarded as a censored event, suggesting that HSCT may abrogate the adverse impact of s‐AML on survival to a certain extent (Table S4).

**Table 4:** Multivariate analyses for CR, ED, EFS, and OS^a^

	CR		ED		EFS		OS	
Covariate	OR (95% CI)	*P*	OR (95% CI)	*P*	HR (95% CI)	*P*	HR (95% CI)	*P*
Total								
Age (y)	0.945 (0.926‐0.963)	<.001	1.061 (1.031‐1.093)	<.001	1.040 (1.029‐1.052)	<.001	1.042 (1.030‐1.055)	<.001
WBC (×10^9^/L)		NS		NS	1.004 (1.002‐1.007)	<.001	1.005 (1.003‐1.008)	<.001
Cytogenetics^b^	0.328 (0.177‐0.607)	<.001		NS	3.074 (2.159‐4.377)	<.001	3.441 (2.371‐4.994)	<.001
s‐AML vs de novo AML		NS		NS		NS	2.023 (1.195‐3.426)	.009
*FLT3*‐ITD	0.433 (0.224‐0.837)	.013		NS	1.669 (1.134‐2.458)	.009	1.957 (1.297‐2.955)	.001
*KMT2A*‐PTD	0.330 (0.131‐0.830)	.018		NS		NS	1.780 (1.000‐3.167)	.05
*NRAS*		NS	3.010 (1.368‐6.620)	.006		NS		NS
Bi*CEBPA*	7.004 (1.647‐29.782)	.008		NS	0.435 (0.274‐0.689)	<.001	0.333 (0.183‐0.606)	<.001
Elderly								
BM blasts (%)		NS	1.050 (1.016‐1.085)	.004		NS	1.015 (1.000‐1.030)	.051
WBC (×10^9^/L)		NS		NS	1.009 (1.002‐1.016)	.01	1.008 (1.001‐1.016)	.033
Cytogenetics^b^		NS		NS	3.370 (1.642‐6.916)	.001	2.693 (1.241‐5.844)	.012
*CBFβ‐MYH11*		NS	49.197 (3.050‐793.497)	.006	4.171 (1.444‐12.048)	.008	6.441 (2.097‐19.783)	.001
*KMT2A*‐PTD	0.233 (0.055‐0.987)	.048		NS		NS		NS
*IDH1*		NS		NS	6.918 (1.559‐30.698)	.011		NS
Secondary								
Age (y)	0.927 (0.870‐0.987)	.018		NS	1.073 (1.020‐1.129)	.007		NS
HB (g/L)	1.032 (1.004‐1.061)	.027		NS		NS		NS
WBC (×10^9^ /L)		NS		NS	1.018 (1.005‐1.031)	.006	1.014 (1.003‐1.026)	.017
Cytogenetics^b^		NS		NS	5.455 (1.621‐18.354)	.006	5.547 (1.789‐17.193)	.003
*NRAS*		NS	13.125 (1.662‐103.673)	.015	7.321 (1.700‐31.521)	.008	4.104 (1.096‐15.368)	.036

Abbreviation: AML, acute myeloid leukemia; BM, bone marrow; CR, complete remission; ED, early death; EFS, event‐free survival; HB, hemoglobin; HR, hazard ratio; OR, odds ratio; OS, overall survival; WBC, white blood cell.

a Patients who received HSCT were censored at the time of HSCT.

b Unfavorable vs others.

## 4 DISCUSSION

Acute myeloid leukemia is a hematologic malignancy with a relative high incidence rate especially in high Human Development Index (HDI) countries.^13^ The incidence of AML increases with age, which makes AML a tumor of the elderly population.^1^ As a separate type of AML, s‐AML becomes more and more common due to the aging population and the increasing use of leukemogenic cytotoxic therapy.^14^

Our study demonstrated that e/s‐AML patients have distinct clinical features compared with young de novo AML patents, such as lower WBC and BM blasts at diagnosis, which indicate that both elderly and secondary AML are less proliferative diseases, partly because they may have either transformed from MDS or experienced an undetected MDS period. Consistently, R. Coleman Lindsley et al^15^ reported that one third elderly de novo AML and t‐AML patients carried “secondary‐type” mutations and showed clinical characteristics indistinguishable from AHD‐AML, indicating that a large proportion of elderly de novo AML and t‐AML patients may transit through unconscious myelodysplastic disease.

The genetic and molecular heterogeneity of AML have been widely acknowledged and integrated to optimize the prediction of clinical outcomes for AML patients. Previous studies demonstrated that *FLT3*‐ITD, *TP*53, *RUNX1, ASXL1* aberrations, and *KMT2A* rearrangements are associated with adverse prognosis, while patients with bi*CEBPA* mutations, *RUNX1‐RUNXT1T1*, and *CBFβ‐MYH11* seem to have a relatively good outcome.^10^, ^16^, ^17^, ^18^, ^19^, ^20^, ^21^, ^22^ However, our knowledge concerning distribution and prognostic significance of gene alterations in e/s‐AML patients remains scarce. Our study indicated that elderly and secondary patients carried more inferior molecular events such as *KMT2A‐*AF9 and *DNMT3A* mutations and less favorable ones including *RUNX1‐RUNXT1T1*, *CBFβ‐MYH11*, and biallelic *CEBPA.* Furthermore, genetic aberrations including *NRAS*, *DNMT3A*, *IDH1* mutations, and *CBFβ‐MYH11* conveyed prognostic information independently in e‐AML or s‐AML patients. However, some significant genetic alterations such as *TP53*, *TET2*, *ASXL1*, and *RUNX1* mutations were not routinely tested in our center and accordingly not available in this retrospective study. Tsai et al^22^ reported that the e‐AML harbored more mutations concerning *PTPN11*, *NPM1*, *RUNX1*, *ASXL1*, *TET2*, *DNMT3A*, and *TP53* genes, but had less *WT1* mutations. In addition, *DNMT3A* and *TP53* mutations were independent adverse prognostic factors for elderly patients. Other studies^10^, ^23^, ^24^, ^25^ showed epigenetic modifier genes (EMGs) including *DNMT3A*, *ASXL1*, and *TET2* were more frequent in e‐AML patients, which was thought to be associated with age‐related clonal hematopoiesis and inferior survival. S‐AML patients were reported to carry less *NPM1* mutations and *FLT3*‐ITD.^26^, ^27^ Besides, patients with AML secondary to MDS and CMML carried more *ASXL1* and *NRAS* mutations.^27^ Currently, a prospective study including more molecular events is performed in our center, which will provide more integrated results concerning the distribution and prognostic significance of molecular alterations in e/s‐AML patients.

Consistent with previous studies,^3^, ^6^, ^24^, ^28^ we observed that both e‐AML and s‐AML were associated with lower CR rate and a short duration of EFS and OS. Although some new therapeutic agents were applied to these high‐risk AML patients, the treatment of e/s‐AML remains a challenge, and there is no consensus on this controversial issue. Some studies indicated that because of remarkable improvement in supportive care, intensive therapy leads to a better survival without increasing early death rate in e/s‐AML patients.^3^, ^6^, ^29^, ^30^ Canadian Consensus Guidelines recommended that patients under the age of 80 should be treated with intensive therapy, except for those with major comorbidities or adverse risk cytogenetics who are not candidates for HSCT.^31^ However, other studies including MD Anderson reported that patients receiving less intensive therapy such as hypomethylation drugs had superior prognosis compared with those receiving intensive induction.^28^, ^32^, ^33^ Our study showed that e/s‐AML patients treated with intensive therapy had a higher CR rate and tended to have a lower frequency of positive MRD. More importantly, a tendency of a longer EFS and OS was observed in intensively treated patients compared with those who received hypomethylation therapy or palliative treatment. These results may partially be because patients receiving intensive therapy have better performance status and fewer comorbidities, and our prospective study will provide more convincing evidence.

Recently, a study reported that CPX‐351 could improve the response rates and survival of patients aged 60 to 75 with s‐AML compared with standard 3 + 7 treatment.^34^ The Food and Drug Administration (FDA) approved glasdegib and venetoclax for the treatment of patients over 75 years old, or young patients who have comorbidities that are not suitable for intensive induction chemotherapy.^35^, ^36^ We expect that the frontline use of these new drugs may improve the outcomes of e/s‐AML individuals, which need to be compared with traditional intensive therapy in prospective research.

In summary, the incidence of e/s‐AML is increasing and will be more common in the future, which merits our attention. Both elderly and secondary AML presented with distinct clinical, cytogenetic, and molecular features, whose prognosis remains dismal compared with young de novo patients, with a significant shorter EFS and OS. Intensive therapy could improve the prognosis of e/s‐AML patients to a certain degree and should be recommended for patients as long as the conditions are appropriate. HSCT could abrogate the adverse prognostic impact of s‐AML and should be considered for the treatment of fit s‐AML patients. More importantly, prospective clinical trials with new drugs are warranted in this special group of patients.

## CONFLICT OF INTEREST

The authors declare no conflict of interest.

## Supporting information

Table S1. Gene abnormalities and clinical aspects.
Table S2. Univariate analyses for CR and ED.
Table S3. Univariate analyses for event‐free survival and overall survival.
Table S4. Multivariate analysis of OS in total cohort with HSCT not censored.
