# Supplementary material for: Genetic alteration patterns and clinical outcomes of elderly and secondary acute myeloid leukemia
Source: Hematol Oncol. 2019 Aug 20;37(4):456–63. doi: 10.1002/hon.2656 (PMC6899678; doi:10.1002/hon.2656)
Supplement: Supplementary file 1 — Table S1. Gene abnormalities and clinical aspects. Table S2. Univariate analyses for CR and ED. Table S3. Univariate analyses for event‐free survival and overall survival. Table S4. Multivariate analysis of OS in total cohort with HSCT not censored. [file HON-37-456-s001.docx]

| **Supplementary table 1.** Gene abnormalities and clinical aspects. | | | | | |
| --- | --- | --- | --- | --- | --- |
| Gene abnormalities | Median age, y (range) | Median WBC count, × 10^9^/L (range) | Median HB, g/L (range) | Median PLT count, × 10^9^/L (range) | Median BM blasts, % (range) |
| **Elderly** |  |  |  |  |  |
| Bi*CEBPA* |  |  |  |  |  |
| Mutated | 65 (61-71) | 12.18 (2.06-73.00) | 110 (96-142) | 32 (5-171) | 53.5 (18.5-77.5) |
| Unmutated | 65 (60-79) | 10.95 (0.84-214.4) | 81 (15-135) | 52 (3-512) | 61 (18-96.5) |
| P | 0.767 | 0.681 | < 0.001 | 0.200 | 0.211 |
| *DNMT3A* |  |  |  |  |  |
| Mutated | 63 (60-77) | 21.05 (1.28-214.4) | 88.5 (47-131) | 69.5 (5-396) | 60 (20.5-92) |
| Unmutated | 65 (60-79) | 7.51 (0.5-197.81) | 82 (15-142) | 45 (3-512) | 60.5 (18-96.5) |
| P | 0.163 | 0.151 | 0.322 | 0.110 | 0.721 |
| *IDH1* |  |  |  |  |  |
| Mutated | 61 (60-62) | 1.35 (1.28-1.42) | 120 (110-130) | 285 (174-396) | 52.3 (20.5-84) |
| Unmutated | 64 (60-79) | 11.31 (0.99-21.44) | 82 (15-142) | 54 (3-512) | 61 (18.5-96.5) |
| P | 0.083 | 0.005 | 0.038 | 0.016 | 0.772 |
| *IDH2* |  |  |  |  |  |
| Mutated | 65 (60-77) | 3.4 (0.99-75.35) | 84 (59-135) | 70 (12-257) | 61 (24-92) |
| Unmutated | 64 (60-79) | 12.62 (1.28-214.4) | 82.5 (15-142) | 54.5 (3-512) | 60.8 (18.5-96.5) |
| P | 0.701 | 0.009 | 0.593 | 0.698 | 0.800 |
| *NPM1* |  |  |  |  |  |
| Mutated | 66 (60-77) | 20.7 (1.23-142.33) | 82 (47-125) | 54 (11-323) | 61.5 (21.5-96.5) |
| Unmutated | 64 (60-79) | 6.96 (0.84-214.4) | 84.5 (15-142) | 45 (3-512) | 59.5 (18-96) |
| P | 0.156 | 0.037 | 0.548 | 0.167 | 0.096 |
| **Secondary** |  |  |  |  |  |
| *KMT2A*-AF9 |  |  |  |  |  |
| With | 48 (36-61) | 51.21 (1.0-97.15) | 100 (80-121) | 74.5 (20-119) | 73.5 (46.5-95) |
| Without | 59 (22-77) | 12.35 (0.8-144.10) | 69.5 (34-143) | 59 (6-513) | 43.3 (16.5-91.5) |
| P | 0.178 | 0.651 | 0.091 | 0.651 | 0.068 |
| Abbreviation: WBC, white blood cell; HB, hemoglobin; PLT, platelet; BM, Bone marrow. | | | | | |

| **Supplementary table 2.** Univariate analyses for CR and ED. | | | | | | |
| --- | --- | --- | --- | --- | --- | --- |
| Factor | CR | Not CR | P | ED | Not ED | P |
| **Total** |  |  |  |  |  |  |
| Median age, y (range) | 45 (18-77) | 58 (21-81) | < 0.001 | 57 (30-81) | 48 (17-77) | < 0.001 |
| Male gender, n (%) | 256/467 (54.8) | 65/147 (44.2) | 0.025 | 28/68 (41.2) | 314/581 (54.0) | 0.044 |
| Median HB, g/L (range) | 87 (15-171) | 76 (39-143) | 0.002 | 75.5 (46-133) | 84 (15-171) | 0.048 |
| *RUNX1-RUNX1TI* | 62/428 (14.5) | 5/116 (4.3) | 0.003 |  |  | NS |
| *CBFβ-MYH11* |  |  | NS | 5/41 (12.2) | 27/487 (5.5) | 0.170 |
| *FLT3*-ITD | 46/430 (10.7) | 28/114 (24.6) | < 0.001 |  |  | NS |
| *KMT2A* rearrangement |  |  | NS | 5/44 (11.4) | 27/522 (5.2) | 0.171 |
| *KMT2A*-PTD | 17/427 (4.0) | 16/113 (14.2) | < 0.001 | 6/44 (13.6) | 28/521 (5.4) | 0.06 |
| *CKIT* | 49/413 (11.9) | 4/111 (3.6) | 0.01 |  |  | NS |
| *NRAS* |  |  | NS | 11/45 (24.4) | 68/520 (13.1) | 0.035 |
| Bi*CEBPA* | 79/429 (18.4) | 2/115 (1.7) | < 0.001 | 2/45 (4.4) | 81/524 (15.5) | 0.045 |
| *DNMT3A* | 47/428 (11) | 22/114 (19.3) | 0.018 |  |  | NS |
| Cytogenetics^*^ | 45/424 (10.6) | 31/122 (25.4) | <0.001 | 14/54 (25.9) | 69/524 (13.5) | 0.011 |
| **Elderly** |  |  |  |  |  |  |
| Median HB, g/L (range) | 90.5 (15-142) | 76 (46-123) | 0.025 |  |  | NS |
| Median BM blasts,  % (range) |  |  | NS | 70.5  (17.5-96.5) | 56.8  (18-96) | 0.008 |
| *CBFβ-MYH11* |  |  | NS | 3/16 (18.8) | 1/97 (1) | 0.008 |
| *FLT3*-ITD | 8/77 (10.4) | 10/40 (25) | 0.038 |  |  | NS |
| *KMT2A*-PTD | 3/76 (3.9) | 6/40 (15) | 0.08 |  |  | NS |
| Bi*CEBPA* | 10/75 (13.3) | 0/40 (0) | 0.039 |  |  | NS |
| **Secondary** |  |  |  |  |  |  |
| Median age, y (range) | 52 (21-74) | 63 (26-77) | 0.004 |  |  | NS |
| Median WBC count,  × 10^9^/L (range) |  |  | NS | 26.31  (3.24-117.07) | 6.22  (0.8-144.1) | 0.018 |
| Median HB, g/L (range) | 80 (34-143) | 65 (39-119) | 0.067 |  |  | NS |
| Median PLT, × 10^9^/L (range) |  |  | NS | 17.5  (2-116) | 66  (6-752) | 0.001 |
| *NRAS* |  |  | NS | 3/5 (60) | 4/39 (10.3) | 0.023 |
| *IDH2* |  |  | NS | 1/1 (100) | 0/21 (0) | 0.045 |
| Cytogenetics^*^ | 3/27 (11.1) | 6/18 (38.9) | 0.148 | 4/8 (50) | 5/41 (12.2) | 0.043 |
| Abbreviation: CR, complete remission; ED, early death. HB, hemoglobin; WBC, white blood cell; BM, bone marrow; PLT, platelet. | | | | | | |
| ^*^Unfavorable vs. others. | | | | | | |

| **Supplementary table 3.** Univariate analyses for event-free survival and overall survival. | | | | | |
| --- | --- | --- | --- | --- | --- |
|  | EFS | |  | OS | |
| Covariate | HR (95% CI) | P |  | HR (95% CI) | P |
| **Total** |  |  |  |  |  |
| Age (y) | 1.039 (1.030-1.047) | < 0.001 |  | 1.043 (1.033-1.053) | < 0.001 |
| WBC (× 10^9^/L) | 1.003 (1.001-1.005) | 0.003 |  | 1.004 (1.002-1.006) | 0.001 |
| HB (g/L) | 0.992 (0.987-0.996) | < 0.001 |  | 0.992 (0.987-0.997) | 0.001 |
| Cytogenetics^*^ | 2.539 (1.863-3.461) | < 0.001 |  | 2.890 (2.089-3.998) | < 0.001 |
| s-AML vs de novo AML | 1.679 (1.163-2.424) | 0.006 |  | 1.758 (1.185-2.606) | 0.005 |
| *RUNX1-RUNX1TI* | 0.635 (0.423-0.952) | 0.028 |  | 0.637 (0.406-1.000) | 0.05 |
| *CBFβ-MYH11* | 0.413 (0.195-0.877) | 0.021 |  | 0.488 (0.216-1.100) | 0.083 |
| *FLT3-*ITD | 2.089 (1.503-2.904) | < 0.001 |  | 2.343 (1.659-3.309) | < 0.001 |
| *KMT2A* rearrangement | 1.847 (1.156-2.952) | 0.01 |  | 2.341 (1.458-3.760) | < 0.001 |
| *KMT2A-*PTD | 2.001 (1.235-3.241) | 0.005 |  | 2.345 (1.422-3.867) | 0.001 |
| Bi*CEBPA* | 0.338 (0.220-0.521) | < 0.001 |  | 0.243 (0.138-0.427) | < 0.001 |
| *DNMT3A* | 1.614 (1.169-2.226) | 0.004 |  | 1.586 (1.116-2.253) | 0.01 |
| *IDH1* |  | NS |  | 0.526 (0.247-1.123) | 0.097 |
| **Elderly** |  |  |  |  |  |
| Age (y) | 1.056 (1.002-1.113) | 0.043 |  | 1.049 (0.999-1.103) | 0.056 |
| BM blasts (%) |  | NS |  | 1.011 (1.001-1.021) | 0.037 |
| WBC (× 10^9^/L) | 1.005 (1.001-1.010) | 0.022 |  | 1.006 (1.002-1.011) | 0.008 |
| HB (g/L) | 0.989 (0.980-0.998) | 0.012 |  | 0.988 (0.979-0.998) | 0.013 |
| Cytogenetics^*^ | 2.066 (1.154-3.697) | 0.015 |  | 2.269 (1.262-4.079) | 0.006 |
| *RUNX1-RUNX1TI* | 0.507 (0.204-1.259) | 0.143 |  |  | NS |
| *CBFβ-MYH11* | 3.114 (1.124-8.627) | 0.029 |  | 3.314 (1.193-9.204) | 0.022 |
| *FLT3-*ITD | 2.066 (1.170-3.648) | 0.012 |  | 2.409 (1.353-4.291) | 0.003 |
| Bi*CEBPA* | 0.315 (0.099-1.000) | 0.05 |  | 0.395 (0.124-1.258) | 0.116 |
| *DNMT3A* |  | NS |  | 0.602 (0.323-1.122) | 0.11 |
| *IDH1* | 3.823 (0.909-16.088) | 0.067 |  |  | NS |
| *IDH2* |  | NS |  | 0.400 (0.180-0.890) | 0.025 |
| **Secondary** |  |  |  |  |  |
| Age (y) | 1.021 (0.993-1.050) | 0.147 |  | 1.026 (0.994-1.058) | 0.109 |
| WBC (× 10^9^/L) | 1.011 (1.003-1.019) | 0.005 |  | 1.012 (1.003-1.020) | 0.005 |
| HB (g/L) | 0.989 (0.975-1.003) | 0.127 |  | 0.990 (0.976-1.005) | 0.19 |
| Cytogenetics^*^ | 3.208 (1.363-7.555) | 0.008 |  | 3.589 (1.499-8.593) | 0.004 |
| *FLT3-*ITD | 2.607 (0.735-9.244) | 0.138 |  |  | NS |
| *NRAS* | 3.029 (0.934-9.826) | 0.065 |  | 3.415 (1.030-11.33) | 0.045 |
| *DNMT3A* | 1.878 (0.783-4.505) | 0.158 |  |  | NS |
| Abbreviation: EFS, event-free survival; OS, overall survival; WBC, white blood cell; HB, hemoglobin; BM, bone marrow; HR, hazard ratio. | | | | | |
| ^*^Unfavorable vs. others. | | | | | |

| **Supplementary table 4.** Multivariate analysis of OS in total cohort with HSCT not censored. | | |
| --- | --- | --- |
|  | OS (HSCT not censored) |  |
| Covariate | HR (95% CI) | P |
| **Total** |  |  |
| Age (y) | 1.045 (1.034-1.057) | < 0.001 |
| WBC (× 10^9^/L) | 1.004 (1.002-1.006) | < 0.001 |
| Cytogenetics^*^ | 2.802 (1.987-3.951) | < 0.001 |
| s-AML vs de novo AML |  | NS |
| *FLT3-*ITD | 1.730 (1.195-2.505) | 0.004 |
| *KMT2A-*PTD |  | NS |
| Bi*CEBPA* | 0.365 (0.207-0.644) | 0.001 |
| Abbreviation: OS, overall survival; HSCT, hematopoietic stem cell transplantation; WBC, white blood cell; | | |
| HR, hazard ratio. | | |
| ^*^Unfavorable vs. others. | | |
